# Supplementary material for: The fungus Leptosphaerulina persists in Anopheles gambiae and induces melanization
Source: PLoS One. 2021 Feb 22;16(2):e0246452. doi: 10.1371/journal.pone.0246452 (PMC7899377; doi:10.1371/journal.pone.0246452)
Supplement: S1 Fig — (A) A graphical representation of fungus introduction to anopheles immature stages. To establish fungus free mosquitoes for infection with Leptosphaerulina sp., insectary eggs from adults harboring diverse fungi (B, panel W screened with ITS1/ITS4 primers expected size 580bp) were washed with 1% HCl for two generations and confirmed fungus negative with ITS1/4 primers (B, panel X). Uninfected mosquitoes were infected and confirmed to harbor Leptosphaerulina sp. at larvae (L), pupae (P), and adult (A) developmental stages using ITS1/4 primers (B, panel Y, culture isolate [CL] included as positive control while uninfected included as controls). These were confirmed as Leptosphaerulina sp. by rescreening infected and uninfected samples using Lepto521F/896R primers developed from isolate whole genome sequence (B, panel Z with expected size of 320bp). (C) The rate of emergence were relatively lower in fungus infected Anopheles gambiae. (DOCX) [file pone.0246452.s001.docx]

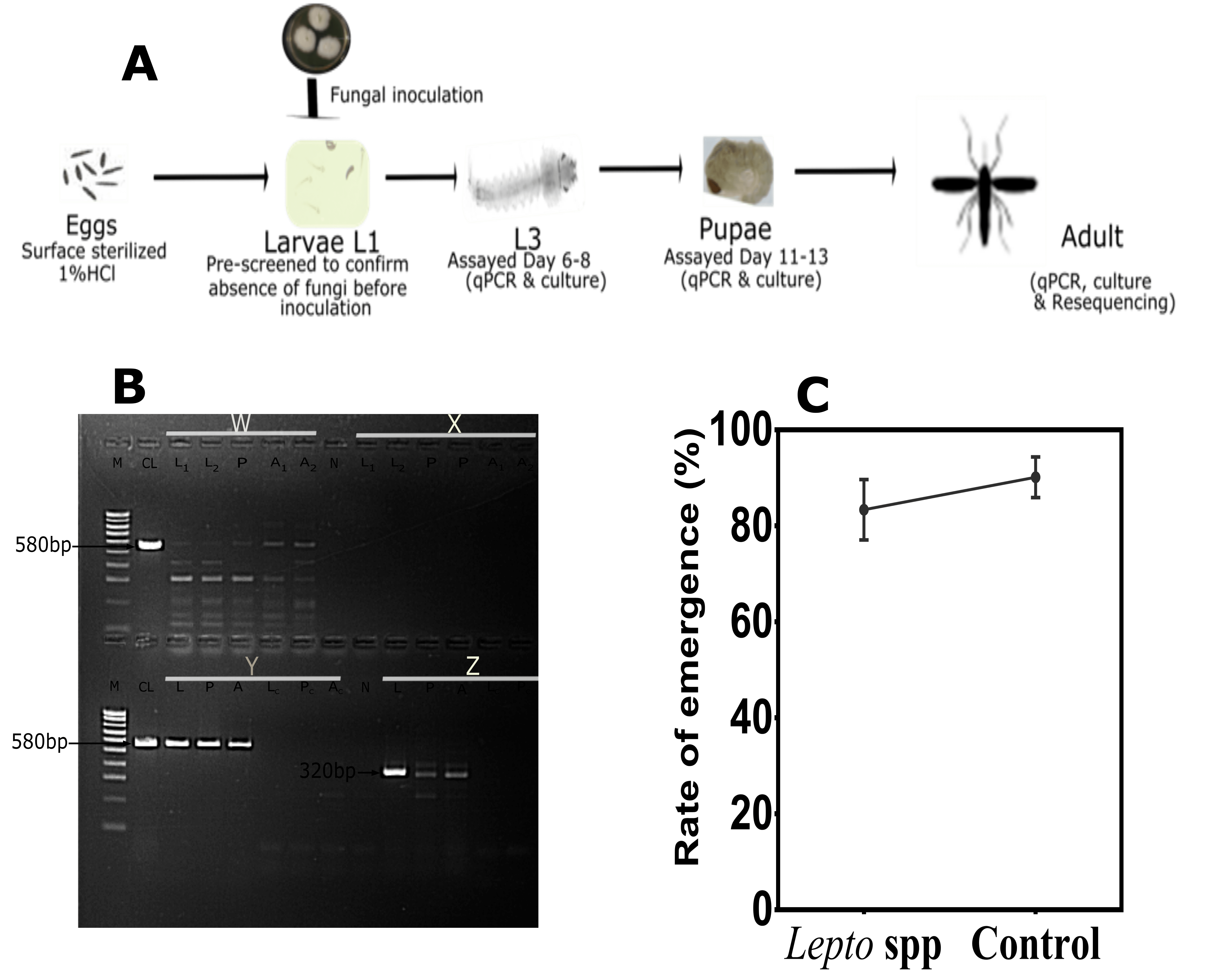


**S1 Fig: Establishment of fungus in *Anopheles gambiae***. (**A)** a graphical representation of fungus introduction to anopheles immature stages. To establish fungus free clean mosquitoes for infection with *Leptosphaerulina sp*, insectary eggs from adults harboring diverse fungi (**B,** panel **W** screened with ITS1/ITS4 primers expected size 580bp) were washed with 1% HCl for two generations and confirmed fungus negative with ITS1/4 primers (**B**, panel **X**). Clean mosquitoes were infected and confirmed to harbor *Leptosphaerulina sp* at larvae (*L*), pupae (*P*), and adult (*A*) developmental stages using ITS1/4 primers (**B**, panel **Y**, culture isolate [CL] included as positive control while uninfected included as controls). These were confirmed as Leptosphaerulina sp by rescreening infected and uninfected samples using Lepto521F/896R primers developed from isolate whole genome sequence (**B**, panel **Z** with expected size of 320bp). (**C**) The rate of emergence were relatively lower in fungus infected *Anopheles gambiae.*
