# Supplementary material for: The fungus Leptosphaerulina persists in Anopheles gambiae and induces melanization
Source: PLoS One. 2021 Feb 22;16(2):e0246452. doi: 10.1371/journal.pone.0246452 (PMC7899377; doi:10.1371/journal.pone.0246452)
Supplement: S2 Fig — The isolate is characterized by white colonies (viewed from culture surface) that form septate-hyphae and spore when stained. Pure isolates obtained from sub-cultured isolates of the midgut dissection were maintained on Saboroud Dextrose Agar media (A is top view of culture on a plate, B is the bottom view). Cultures were stained in Lactophenol blue stain for morphological visualization under microscope (C). (DOCX) [file pone.0246452.s002.docx]

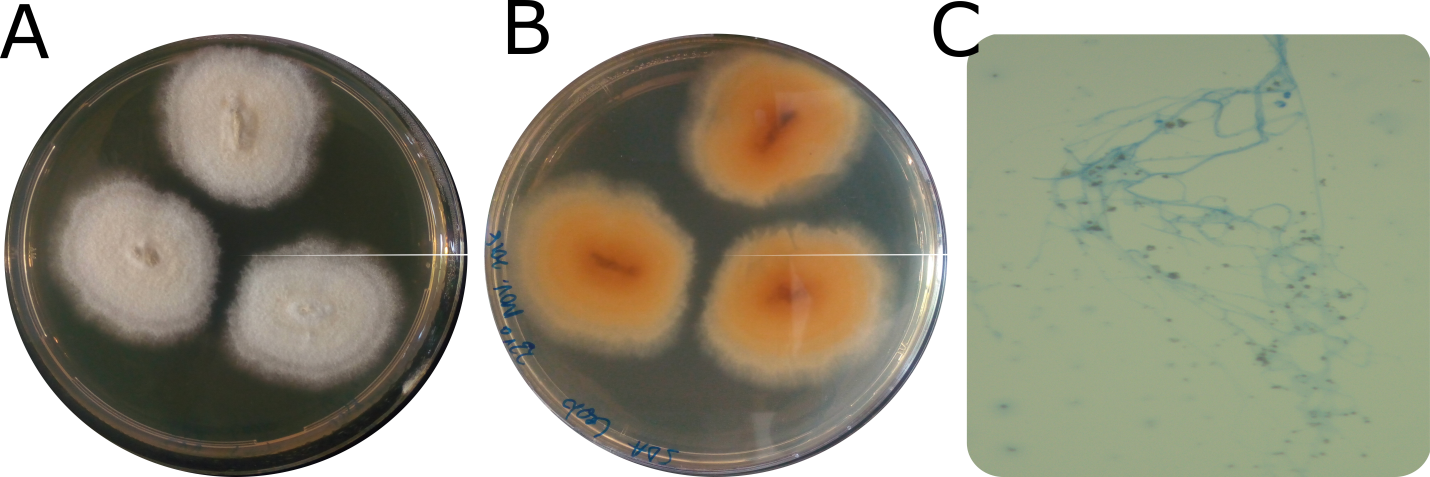


**S2 Fig: Morphology of *Leptosphaerulina*** **sp fungus isolated from semi-field mosquitoes.** The isolate is characterized by white colonies (viewed from culture surface) that form septate-hyphae and spore when stained**.** Pure isolates obtained from sub-cultured isolates of the midgut dissection were maintained on Saboroud Dextrose Agar media (**A** is top view of culture on a plate, **B** is the bottom view). Cultures were stained in Lactophenol blue stain for morphological visualization under microscope (**C**).
