# Supplementary material for: The fungus Leptosphaerulina persists in Anopheles gambiae and induces melanization
Source: PLoS One. 2021 Feb 22;16(2):e0246452. doi: 10.1371/journal.pone.0246452 (PMC7899377; doi:10.1371/journal.pone.0246452)
Supplement: S3 Fig — Relative densities of Leptosphaerulina sp in field Mwea samples at larvae (A, consisting of 25 pools of five) and adult (B, made of 12 pools of 5) stages were assayed using qPCR-HRM and expressed as the ration of Lepto521f/896r fungal gene against host ribosomal gene. (DOCX) [file pone.0246452.s003.docx]

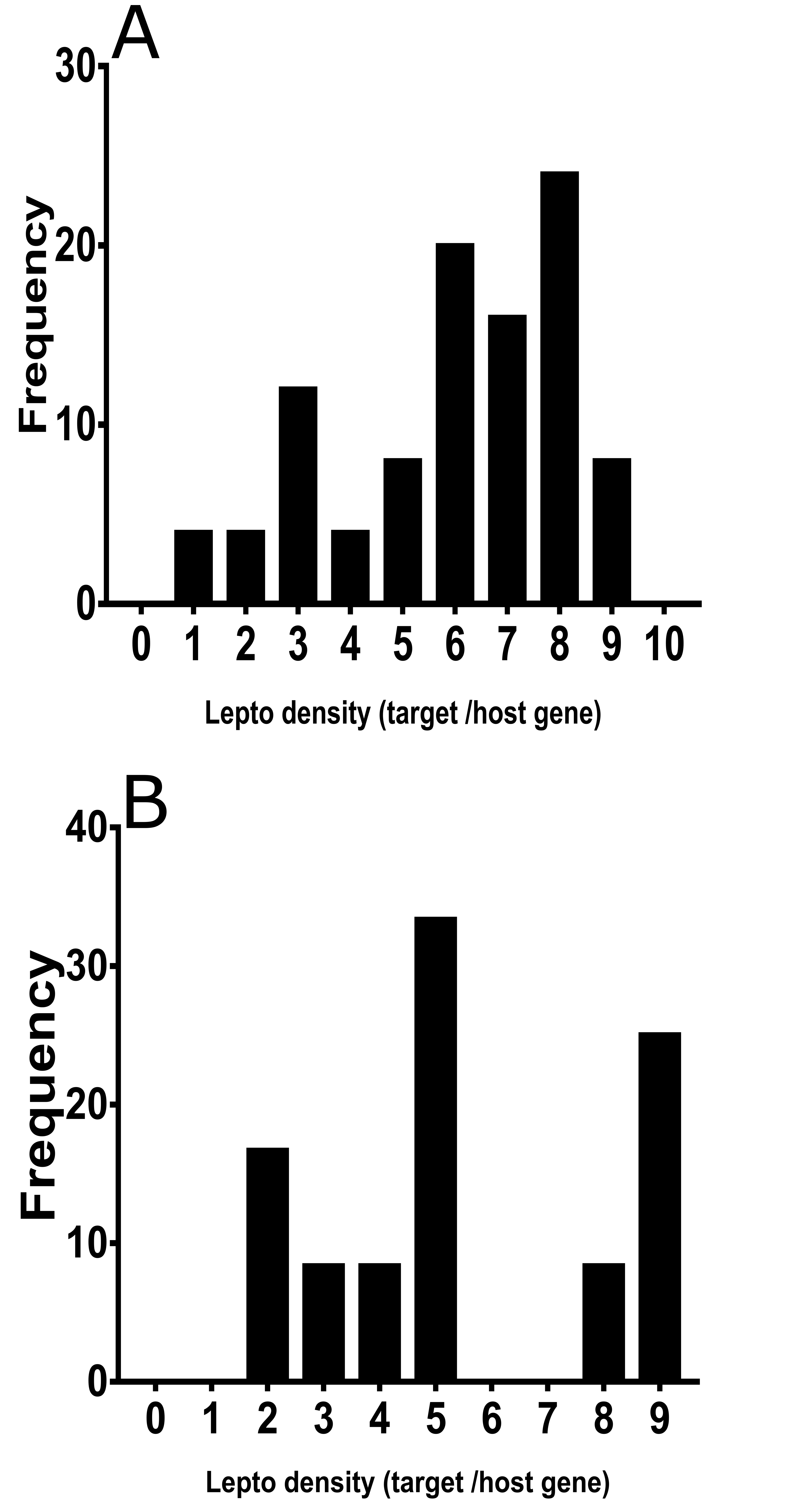


**S3 Fig: Variation of *Leptosphaerulina* sp densities in field caught samples from Mwea location.** Relative densities of *Leptosphaerulina sp* in field Mwea samples at larvae (**A**, consisting of 25 pools of five) and adult (**B**, made of 12 pools of 5) stages were assayed using qPCR-HRM and expressed as the ration of Lepto521f/896r fungal gene against host ribosomal gene.
