# Supplementary material for: The fungus Leptosphaerulina persists in Anopheles gambiae and induces melanization
Source: PLoS One. 2021 Feb 22;16(2):e0246452. doi: 10.1371/journal.pone.0246452 (PMC7899377; doi:10.1371/journal.pone.0246452)
Supplement: S4 Fig — (A) Persistence of the isolate in F1 females did not lower their fertility (Kruska-Wallis test: p = 0.291). (B) Fungus densities in the F1 infected female and male whole mosquitoes’ offspring were assayed by qPCR in 10 day old offspring, and it was noted that intensity of infection burden was relatively high in female (Mann–Whitney test = 1054, p = 0.0003). (C) These densities were distributed in major tissues with increased burden in the midgut (Kruskal-Wallis test, p = 0.0056). The mean bar represents mean±S.E.M., while ** represent p value of <0.01, ***p <0.001, and ****p < 0.0001, ns = not significant (p>0.05). (DOCX) [file pone.0246452.s004.docx]

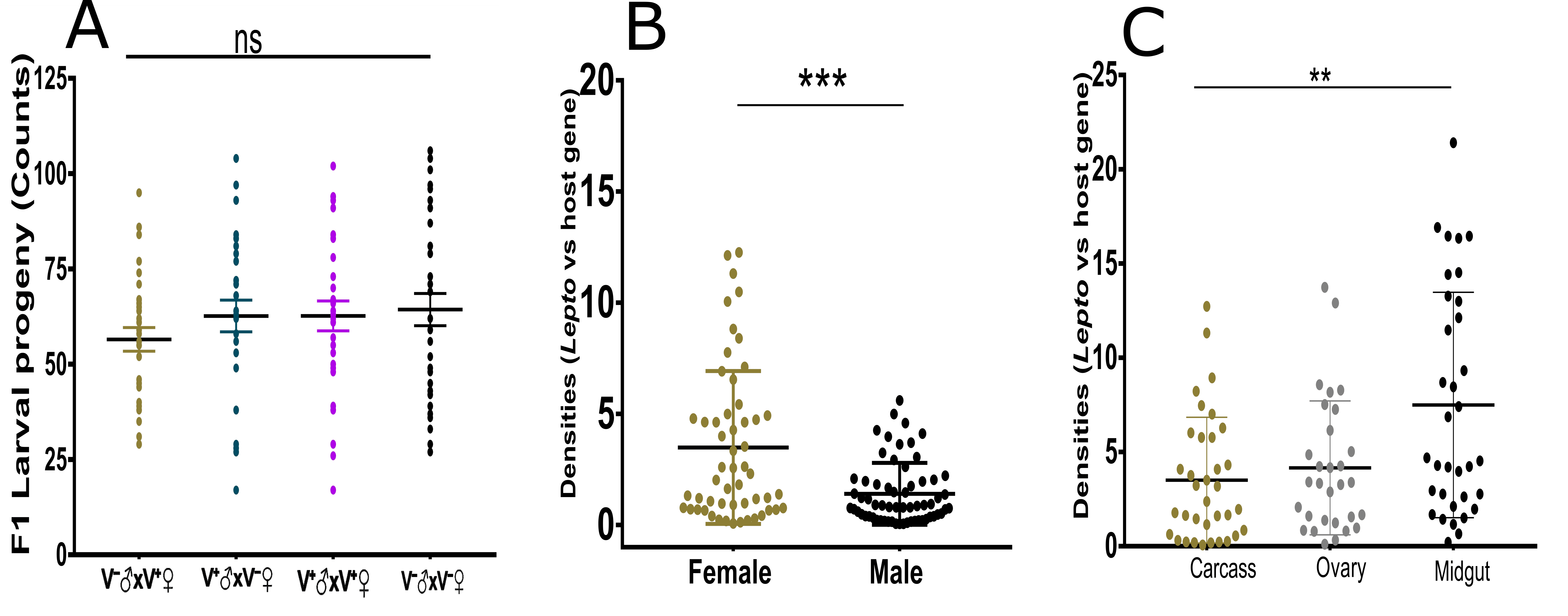


S4 Fig: *Leptosphaerulina* sp infection on *An. gambiae* F1 progeny. (A) Persistence of the isolate in F1 females did not lower their fertility (Kruska-Wallis test: *p*=0.291). (B) Fungus densities in the F1 infected female and male whole mosquitoes’ offspring were assayed by qPCR in 10 day old offspring, and it was noted that intensity of infection burden was relatively high in female (Mann –Whitney test = 1054, p=0.0003). (C) These densities were distributed in major tissues with increased burden in the midgut (Kruskal-Wallis test, *p =* 0.0056). The mean bar represents mean±S.E.M, while ** represent p value of <0.01, ***p <0.001, and ****p < 0.0001, ns = not significant (*p>*0.05).
