# Supplementary material for: The fungus Leptosphaerulina persists in Anopheles gambiae and induces melanization
Source: PLoS One. 2021 Feb 22;16(2):e0246452. doi: 10.1371/journal.pone.0246452 (PMC7899377; doi:10.1371/journal.pone.0246452)
Supplement: S1 Table — (DOCX) [file pone.0246452.s005.docx]

S1 Table: List of**oligonucleotide primers utilized in the amplification experiments**

|  | **Real-Time QRT-PCR primers used to quantify specific transcripts** | | | |
| --- | --- | --- | --- | --- |
| Accession no. | Gene acronym | Primer Sequences (5’ 🡪 3’) | References | |
| M27607 | ITS1 | TCCGTAGGTGAACCTTGCGG | (1) | |
|  | ITS4 | TCCTCCGCTTATTGATATGC |  | |
| MT433814 | Lepto379F | CGAGCGTCATCTACACCCTC | This study | |
|  | Lepto566R | CCCTACCTGATCCGAGGTCA |  | |
| MT433814 | Lepto521F | CCACTGAGGTCAAGTCCGTC | This study | |
|  | Lepto896R | CACATGGGCTTGGAGGGAAT |  |  |
| AGAP010592 | S7_F  S7_R | TCCTGGAGCTGGAGATGAAC  GACGGGTCTGTACCTTCTGG | (2) | |
|  | Oligos for Immunity Assay | | | |
| AGAP005203 | PGRP - LCF  PGRP - LCF | AGAATACCACACTAAGGCACAGT  AGACTTACGATCCTGGTAAATGT | | (3) |
| AGAP000693 | Cecropin_1F | CCAGAGACCAACCAACCACCAA | | (2) |
|  | Cecropin_1R | CGACTGCCAGCACGACAAAGA | |  |
| AGAP011197 | FBN9F | CCAAGATGTCGGGCAAGTAT | | (2) |
|  | FBN9R | TTGTGGTACGTCAGCGAGTC | |  |
| AGAP010815 | TEP1F | ATGCTCTGCTGTCGTTTGTG | | (2) |
|  | TEP1R | TTCGTGTCCTCCGGTATTTC | |  |
| AGAP006348 | LRIM1F | CATCCGCGATTGGGATATGT | | (2) |
|  | LRIM1R | CTTCTTGAGCCGTGCATTTTC | |  |
| AGAP004581 | HSP70F | ACGCCAACGGTATTCTGAAC | |  |
|  | HSP70R | ACAGTACGCCTCGAGCTGAT | | (4) |
| AGAP008212 | CP6M2F | AGGTGAGGAGAGTCGACGAA | |  |
|  | CP6M2R | ATGACACAAACCGACAAGG | |  |
| AGAP011294 | Defensin_1F | GCGGTTCCAAAGTTCCGACA | |  |
|  | Defensin_1R | AGCGGGACACAAAATTGTTC | | (2) |
| AGAP006747 | Rel2F | CGGAGAAGTCGAAGAAAACG | |  |
|  | Rel2R | CACAGGCACACCTGATTGAG | | (2) |

Supporting References

1. White T, Bruns T, S L, Taylor J. Amplication and direct sequencing of fungal ribosomal RNA genes for phylogenetics. In: Innis M, Gelfand D, Sninsky J, White T, editors. PCR Protocols: A Guide to Methods and Applications. 1990. 315–322 p.

2. Bahia AC, Dong Y, Blumberg BJ, Mlambo G, Tripathi A, Chandra R, et al. Exploring Anopheles gut bacteria for Plasmodium blocking activity. Env Microbiol. 2014;16(9):2980–94.

3. Meister S, Agianian B, Turlure F, Relógio A, Morlais I, Kafatos FC, et al. Anopheles gambiae PGRPLC-mediated defense against bacteria modulates infections with malaria parasites. PLoS Pathog. 2009;

4. Sim C, Hong YS, Tsetsarkin KA, Vanlandingham DL, Higgs S, Collins FH. Anopheles gambiae heat shock protein cognate 70B impedes o’nyong-nyong virus replication. BMC Genomics. 2007;8:1–12.
